# RPL39 Was Associated With Sex Differences in Pulmonary Arterial Hypertension

**DOI:** 10.1155/carj/7139235

**Published:** 2025-01-28

**Authors:** Haixia Wang, Ling Li, Guangyuan Zhou, Lu Wang, Zeang Wu

**Affiliations:** ^1^National Health Commission Key Laboratory of Prevention and Treatment of Central Asia High Incidence Diseases (Co-Construction), Department of Scientific Research, The First Affiliated Hospital of Shihezi University, Shihezi, Xinjiang, China; ^2^Department of Preventive Medicine, Shihezi University Medical School Shihezi, Xinjiang, China; ^3^Department of Pathophysiology, School of Basic Medicine, Tongji Medical College, Huazhong University of Science and Technology, Wuhan, Hubei, China; ^4^Department of Respiratory and Critical Care Medicine, Miyun Teaching Hospital of Capital Medical University, Beijing, China

**Keywords:** cell proliferation, macrophages, pulmonary arterial hypertension, ribosome pathway, RPL39, sex differences

## Abstract

Pulmonary arterial hypertension (PAH) is a malignant cardiovascular disease with a complex etiology, in which several types of cells play important roles. Sex differences in disease susceptibility and survival have been observed in PAH patients, but few studies have analyzed the effect of changes in cell type and number on sex differences in PAH at the single-cell level. In this study, we performed a series of analyses on GSE169471 and GSE228644 datasets and found significant changes in the ratio of several types of cells in male PAH lung tissues. Surprisingly, we found that the ratio of macrophages in male PAH samples was 7 times higher than that in females. Consistently, the ratio of M1 macrophages was also significantly increased in male PAH samples. The different expression genes (DEGs) in macrophages were mainly involved in the ribosome pathway, which is closely related to cell proliferation. Inhibition of ribosomal protein L39 (RPL39), a core gene in the ribosome pathway, can inhibit macrophage proliferation and attenuate the sex differences in PAH. In conclusion, our study suggests that ribosome pathway–associated cell proliferation of macrophages might be associated with sex differences in PAH.

## 1. Introduction

Pulmonary arterial hypertension (PAH) is a severe disease that can lead to right heart failure and even death [1, 2]. PAH patients exhibit significant sex differences in disease susceptibility, hemodynamic characteristics, right ventricular function, drug treatment response, and survival rate [3–5]. Several studies have suggested that sex hormones may play a crucial role in the sex differences observed in PAH due to their cardioprotective effect [6–10]. Although some studies have made progress in understanding the sex differences in PAH, such as the role of sex hormones [11–13], further research is necessary to support the clinical provision of gender-based treatment for PAH.

Several studies have suggested that nonhormonal mechanisms may contribute to the sex differences observed in PAH [14, 15]. Various cell types, including endothelial cells (ECs) [16, 17], macrophages [18, 19], smooth muscle cells (SMCs) [20, 21], fibroblasts [22], and T lymphocytes [23], have been found to be involved in the pathogenesis of PAH at the organ and tissue levels. However, limited single-cell level studies have identified specific EC subgroups of PAH patients with different gene expression profiles compared to normal samples [24, 25]. To date, no studies have investigated changes in various types of cells in different gender PAH and normal samples, or changes in gene expression and signal pathways in specific PAH–related cells at the single-cell level.

Here, we utilized single-cell sequencing data to analyze the changes in various types of cells between male and female PAH samples. We conducted a series of analyses to investigate the role of different expressed genes (DEGs) in macrophages between male and female PAH. Finally, we identified one key gene (ribosomal protein L39 [RPL39]) that is associated with PAH and sex differences, and explored the effect of RPL39 knockdown on the sex differences of PAH. Our research provides a theoretical basis for the clinical gender-based treatment of PAH by exploring the reasons for sex differences in PAH patients at the single-cell level.

## 2. Result

### 2.1. Changes in Various Cell Types Between Male and Female PAH Samples

**Figure 1:**
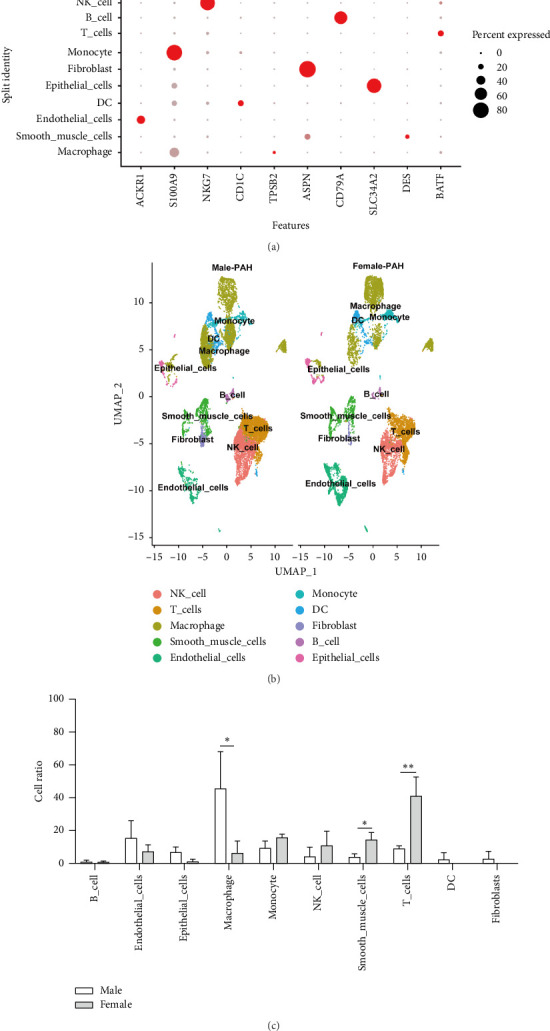
Results of single-cell sequencing analysis in GSE169471 and GSE228644. (a) The cell markers of all kinds of cell clusters screened by the FindAllMarkers function. (b) The changes in cell types and cell number of lungs between male and female PAH samples were shown in the uniform manifold approximation and projection (UMAP) plot of the merged data, and each color represents a cell cluster. (c) The changes in the ratio of cell types in the lungs of male and female PAH samples. ⁣^∗^*p* < 0.05, ⁣^∗∗^*p* < 0.01, ⁣^∗∗∗^*p* < 0.001, and ⁣^∗∗∗∗^*p* < 0.0001.

In this study, we analyzed three male PAH samples and three female PAH samples from GSE169471 and GSE228644 datasets. After data preprocessing, cells with nFeature_RNA > 200, nFeature_RNA < 2500, and percent.mt < 5 were selected, and we finally obtained 11,012 male PAH cells and 9100 female PAH cells. The initial principal component analysis (PCA) and uniform manifold approximation and projection (UMAP) were performed to reduce dimensionality, resulting in 15 clusters in male samples and 22 clusters in female samples. We screened the markers of each cluster with the FindAllMarkers function and used the SingleR *R* package to annotate each cluster according to the identified marker genes. Finally, we identified 10 different cell subgroups in male and female PAH samples, including natural killer cells, T cells, macrophages, smooth muscle cells, ECs, monocytes, dendritic cells, fibroblast, B cells, and epithelial cells (Figures 1(a), 1(b)). To investigate the role of different cell types in PAH sex differences, the scCODA *R* package [26] was used to calculate the ratio of various types of cells in male and female PAH samples. Compared to the male PAH samples, the ratio of SMCs and T cells was significantly increased, and the ratio of macrophages was significantly decreased in female PAH samples (Figure 1(c)). Interestingly, the ratio of macrophages in males was about 7 times higher than that in females, indicating that the changes in the ratio of macrophages between male and female PAH samples may be attributed to sex differences in PAH.

### 2.2. DEGs in Male and Female PAH Macrophages Were Involved in the Translation Process and Ribosome Pathways

**Figure 2:**
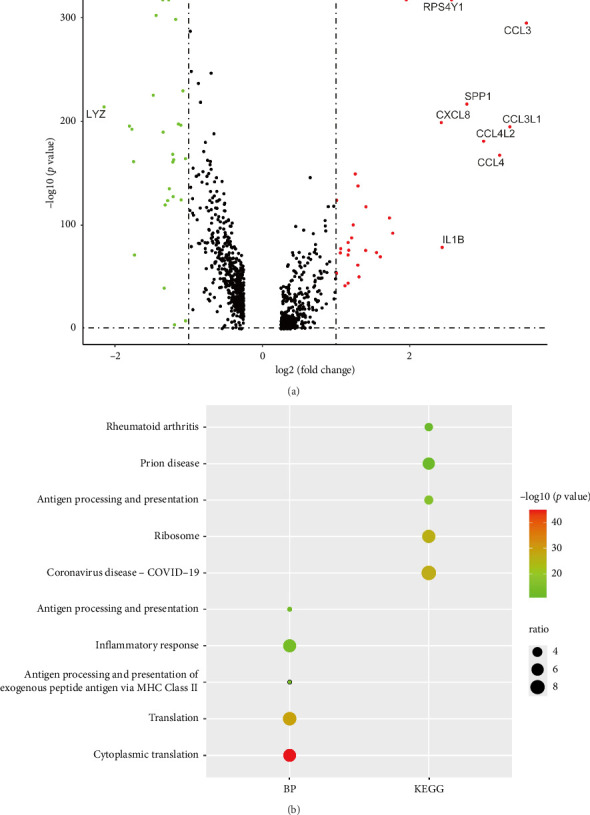
GO and KEGG analyses of DEGs in macrophages between male and female PAH samples. (a) Volcano plot showed the gene expression pattern of all genes, and genes with |log_2_^FC^| > 2 were shown in the plot, male PAH versus female PAH samples, and the color of red, gray, and green represent upregulated, not significantly changed, and downregulated genes. (b) The main biological process and KEGG pathways enriched for DEGs between male and female PAH samples.

**Figure 3:**
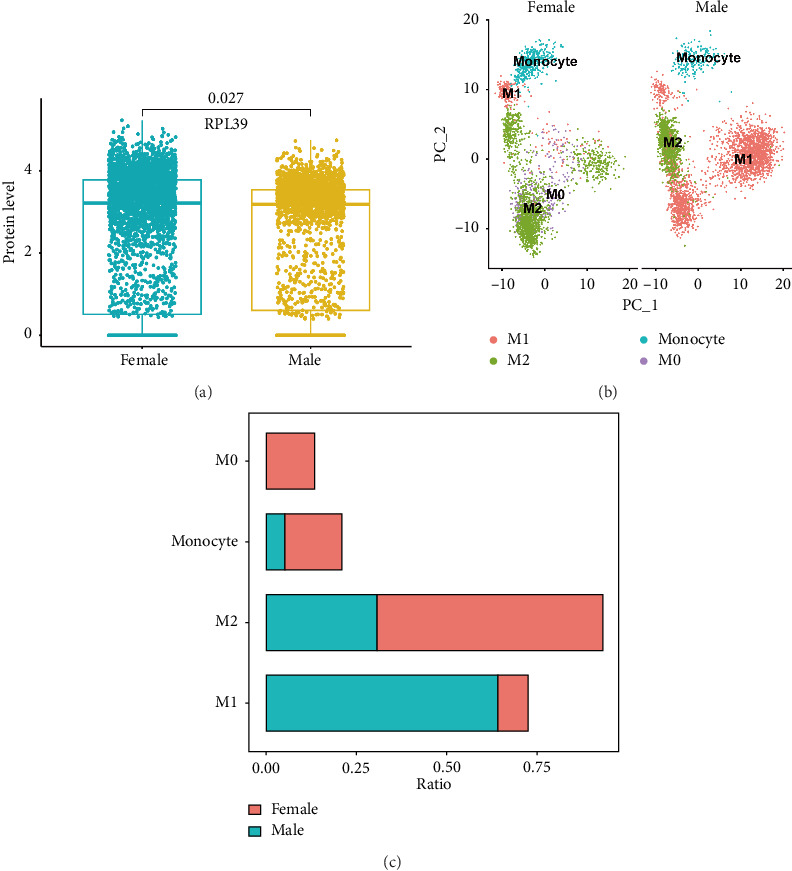
The subgroups of macrophages in male and female PAH samples. (a) The gene expression level of RPL39 between male and female PAH samples. (b) The UMAP plot showed the subgroups of macrophages in male and female PAH samples. (c) The ratio of each subgroup of macrophages in male and female PAH samples.

Genes with *p* value < 0.05 and |log_2_FC| > 1 were screened out as DEGs between male and female PAH macrophages, and we obtained 30 upregulated DEGs and 26 downregulated DEGs, respectively. We showed the expression patterns of all genes in the volcano plot and labeled the gene symbol of DEGs with |log2FC| > 2 in the volcano plot (Figure 2(a)). Gene Ontology (GO) analysis showed that DEGs in male and female PAH macrophages were primarily involved in cytoplasmic translation and translation biological processes. KEGG results indicated that DEGs between male and female PAH macrophages were enriched in the ribosome pathway (Figure 2(b)). Among these DEGs, RPL39 was found to be involved in the ribosome pathway (Figure 3(a)).

### 2.3. The Ratio of M1 Macrophages in Male PAH Samples Was Higher Than That in Female PAH Samples

Studies have suggested that macrophages can be classified into two types based on their functions: proinflammatory M1 macrophages and anti-inflammatory M2 macrophages. In this study, we aimed to analyze the ratio of M1 and M2 macrophages in male and female PAH samples. A total of 6217 macrophages were grouped into 12 cell clusters and annotated as 4 cell subgroups. We found that there were 2334 M1 macrophages, 1119 M2 macrophages, and 184 monocytes in male PAH samples, and 344 monocytes, 344 M0, 213 M1, and 1610 M2 macrophages in female PAH samples (Figure 3(b)). The ratio of M1 macrophages in male PAH samples was higher than that in female PAH samples (64.1% vs. 8.2%). Next, we screened out DEGs of M1 macrophages between male and female PAH samples and 198 DEGs were obtained. These DEGs were enriched in the cytoplasmic translation and ribosome pathway (Table 1). These results suggest that the ribosome pathway may be associated with the changes in the ratio of M1 macrophages between male and female PAH samples.

### 2.4. Knockdown of RPL39 Attenuated Sex Differences in PAH by Inhibiting Macrophages Proliferation

**Figure 4:**
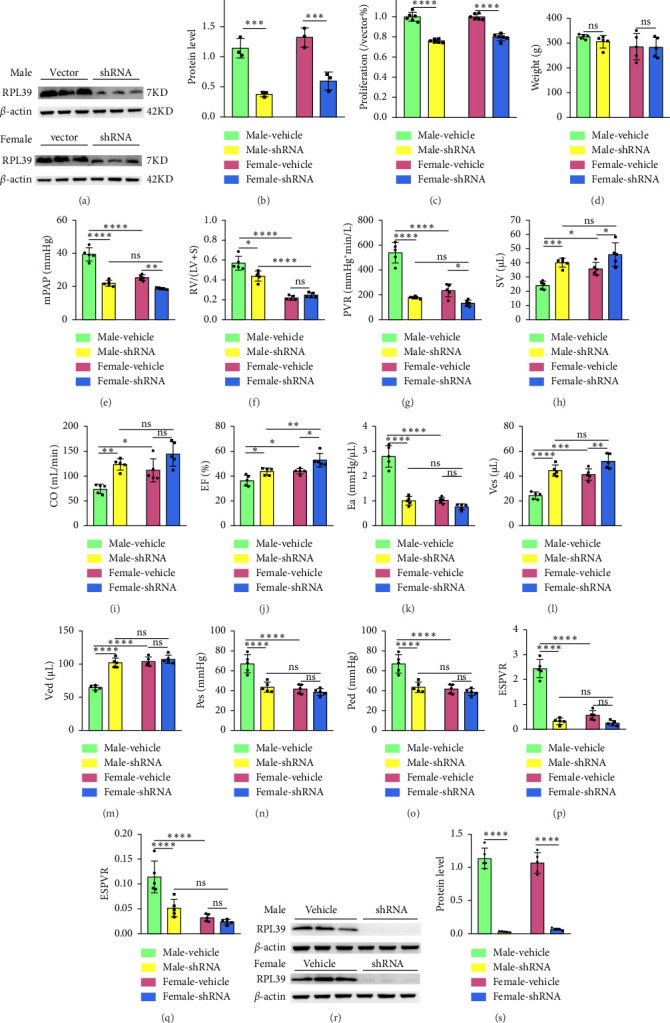
Knockdown RPL39 alleviated PAH by suppressing cell proliferation of macrophages. (a–b) The effect of shRNA on knocking down the protein level of RPL39 in male and female macrophages, shRNA versus vector, *n* = 3; (c) the effect of RPL39 knockdown on the proliferation level of male and female macrophages, shRNA versus vector, *n* = 6; (d) body weight; (e) mean pulmonary arterial pressure, mPAP; (f) right ventricular hypertrophy index; (g) pulmonary vascular resistance, PVR; (h) stroke volume, SV; (i) cardiac output, CO; (j) ejection fraction, EF; (k) effective arterial elastance, Ea; (l) end-systolic volume, Ves; (m) end-diastolic volume, Ved; (n) end-systolic pressure, Pes; (o) end-diastolic pressure, Ped; (p) end-systolic pressure–volume relationship, ESPVR; (q) end-diastolic pressure–volume relationship, EDPVR; (r-s) the protein level of RPL39 in male and female PAH rats with and without shRNA treatment, shRNA versus vehicle, *n* = 5; data in the box plots are presented as mean ± SD. ⁣^∗^*p* < 0.05, ⁣^∗∗^*p* < 0.01, ⁣^∗∗∗^*p* < 0.001, and ⁣^∗∗∗∗^*p* < 0.0001.

**Figure 5:**
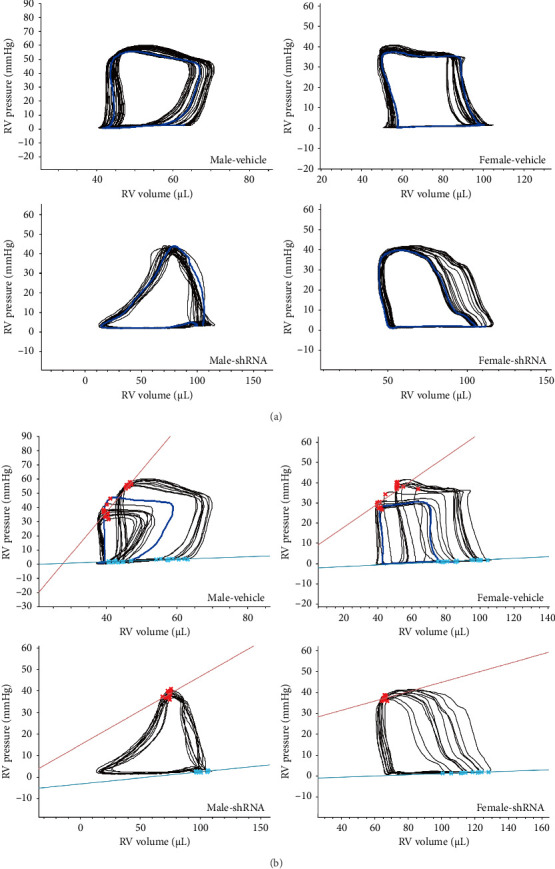
Knocking down RPL39 mitigated the abnormal increase in right ventricular contractility. (a) Right ventricular pressure volume loop (PV-loop) in male-vehicle, male-shRNA, female-vehicle, and female-shRNA groups; the horizontal and vertical coordinates represent the changes of right ventricular volume and pressure from end-systolic to end-diastolic, respectively. (b) Red and blue lines represent the right ventricular systolic PV relationship (ESPVR) and diastolic PV relationship (EDPVR) in male-vehicle, male-shRNA, female-vehicle, and female-shRNA groups. The slopes of ESPVR and EDPVR were positively correlated with right ventricular systolic capacity and diastolic capacity, respectively.

Ribosome signaling pathways have been reported to be involved in cell proliferation, suggesting that inhibition of this pathway could reduce the difference in the ratio of male and female macrophages, thereby attenuating the sex differences in PAH. Interestingly, among these DEGs between male and female PAH macrophages, we found that RPL39 was involved in the ribosome signaling pathway. To investigate the role of RPL39 in PAH, we suppressed its expression with short hairpin RNA (shRNA) and found that RPL39 knockdown significantly inhibited the proliferation of macrophages (Figures 4(a) and 4(c)). At the animal level, RPL39 knockdown significantly decreased the mean pulmonary arterial pressure (mPAP) and pulmonary vascular resistance (PVR) and increased the stroke volume (SV), ejection fraction (EF), and end-systolic volume (Ves) in both male- and female-shRNA groups, compared to male and female PAH groups. In particular, RPL39 knockdown significantly decreased the arterial elastance (Ea), right ventricular hypertrophy index, end-systolic pressure (Pes), end-diastolic pressure (Ped), end-systolic pressure–volume relationship (ESPVR), and end-diastolic pressure–volume relationship (EDPVR), while increasing the cardiac output (CO) and end-diastolic volume (Ved) in male-shRNA group (Figures 4(d), 4(e), 4(f), 4(g), 4(h), 4(i), 4(j), 4(k), 4(l), 4(m), 4(*n*), 4(o), 4(p), 4(q), 4(r), and 4(s)). PV-LOOP results showed that RPL39 knockdown significantly increased the right ventricular end-systolic and Ved and mitigated the abnormal increase in right ventricular systolic capacity in male PAH rats, while RPL39 knockdown only significantly increased the right ventricular Ves in PAH female rats (Figures 5(a) and 5(b)).

**Figure 6:**
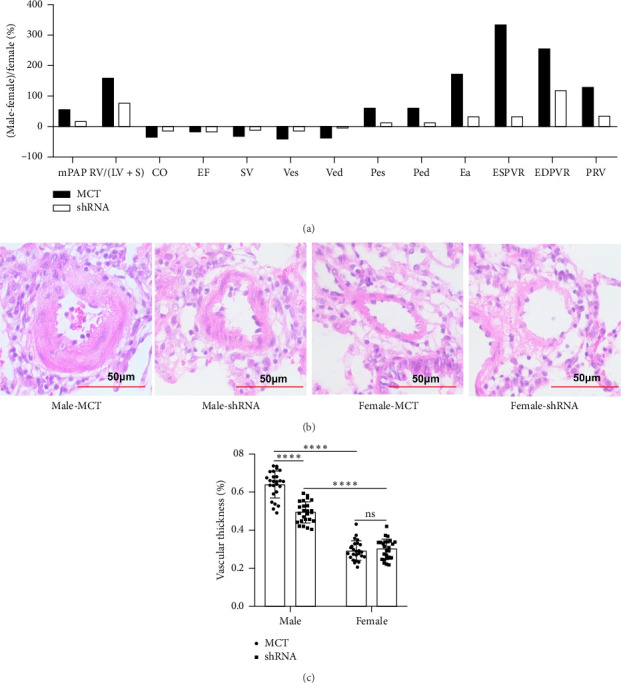
Knockdown RPL39 attenuated the sex differences in PAH. (a) RPL39 knockdown narrowed the degree of difference in hemodynamic parameters between male- and female-shRNA groups. (b–c) RPL39 knockdown narrowed the degree of difference in vascular wall thickness between male- and female-shRNA groups. Data in the box plots are presented as mean ± SD. ⁣^∗^*p* < 0.05, ⁣^∗∗^*p* < 0.01, ⁣^∗∗∗^*p* < 0.001, and ⁣^∗∗∗∗^*p* < 0.0001.

To explore the effect of RPL39 knockdown on sex differences in PAH, we compared the extent of differences in hemodynamic parameters between male and female rats in the PAH and shRNA groups separately. Surprisingly, we found that most hemodynamic parameters in the male PAH group were obviously different from those in the female PAH group, and these differences in hemodynamic parameters were markedly reduced by suppressing the expression of RPL39. For instance, the vascular wall thickness of male PAH samples was 2.18 times higher than that of female PAH samples. However, RPL39 knockdown not only attenuated the increase of vascular wall thickness in the male-shRNA group but also narrowed the degree of difference (to 1.6) in vascular wall thickness between male and female-shRNA groups (Figures 6(a), 6(b), and 6(c)).

## 3. Discussion

Multiple lines of evidence have demonstrated the crucial role of various cell types in the pathogenesis of PAH. For instance, dendritic cells could promote pulmonary vascular remodeling by activating pathogenic T cells and releasing inflammatory factors [27]. In addition, some studies have identified differences in cell types, cell population, gene expression, and signal pathways between PAH and normal samples at the single-cell level [28–30]. However, to our best knowledge, no single-cell studies have been conducted to investigate the role of different cell types in the sex differences of PAH. Our study provides the first evidence that macrophages play an essential role in the sex differences of PAH. In our study, a significantly increased ratio of macrophages in both male and female PAH samples was observed. This finding is consistent with several previous studies that have reported an increased number of macrophages in PAH patients and animals [28, 31]. Moreover, the ratio of macrophages in the male PAH samples was approximately 7 times higher than that in female PAH samples. Notably, the accumulation of macrophages near pulmonary arterioles is a prominent pathological feature of PAH, and there is growing evidence that pulmonary perivascular macrophage–mediated pulmonary inflammation is a key driver of pulmonary remodeling, which leads to elevated right ventricular systolic pressure [32]. Importantly, macrophages are commonly associated with disease severity and progression [33]. Therefore, we speculate that changes in the ratio of macrophages between male and female PAH samples may be related to sex differences in PAH.

M1 macrophages and M2 macrophages play distinct roles in the development of various diseases, and an imbalance of M1/M2 contributed to disease progression [34]. In a hypoxia-induced PH model, an increase in proinflammatory M1 and anti-inflammatory M2 was observed in both bronchoalveolar lavage fluid (BALF) and whole lung samples [35]. One study reported an altered M1/M2 macrophage ratio in the lung tissue of both PAH mice and patients, suggesting that macrophage populations may be a potential therapeutic target for PAH [36]. Interestingly, the ratio of M1 macrophages in male PAH samples was also higher than that in female PAH samples. M1 macrophages amplify inflammation by secreting proinflammatory factors, and the increased M1 macrophages in male PAH samples may be associated with more severe disease severity and worse prognosis.

DEGs in M1 macrophages between male and female PAH samples were mainly involved in the ribosome signaling pathway. Ribosomal proteins play a crucial role in stabilizing ribosomes, DNA repair, apoptosis, drug resistance, growth, proliferation, and chemical resistance [37, 38]. Abnormal expression of ribosomal proteins can cause tumors, metabolic diseases, and autoimmune diseases [39, 40]. Several studies have shown that ribosome biosynthesis is involved in cell growth and division, and ribosome biosynthesis is a classic hallmark of cell proliferation in cancer [41, 42]. In our study, RPL39 was found to play an important role in modulating cell proliferation of macrophages. The similar role of RPL39 in regulating cell proliferation has also been reported in placental trophoblast cells [43], breast cancer cells [44], glioma cells [45], and pancreatic cancer cells [46]. Targeting specific genes in macrophages has been proven to be effective in the treatment of PAH [19, 47]. In this study, we observed that knocking down the expression of RPL39 in macrophages can alleviate PAH. Furthermore, RPL39 knockdown also reduces the sex differences in PAH, partly by decreasing the difference in the ratio of macrophages between male and female PAH samples.

There are several limitations to this study that need to be addressed in future research. First, estrogen is known to be associated with the phenomenon of sex differences in PAH, and this study was unable to exclude the role of estrogen in these differences. Second, although we confirmed that RPL39 knockdown inhibited macrophage proliferation at the cellular level, the changes in macrophage number and ratio in lung tissues of male and female PAH rats after RPL39 knockdown should be determined in future. Third, we used shRNA to reduce the protein level of RPL39 by airway administration, and the effect of shRNA on RPL39 in other cells in lung tissue cannot be excluded. In the future, the CRISPR–Cas9 technology can be used to specifically knockdown RPL39 in macrophages, allowing for a more accurate exploration of the effect of RPL39 in macrophages on sex differences in PAH.

In summary, our study highlights the essential role of macrophages in the sex differences of PAH. The findings suggest that targeting macrophages may be a potential therapeutic strategy for the treatment of PAH.

## 4. Materials and Methods

### 4.1. Data Download, Processing, and Screening

We downloaded single-cell sequencing data of male and female PAH samples from GSE169471 and GSE228644 in the GEO dataset, and patients older than 18 years with a clear diagnosis of PAH and clear gender information were included in the analysis (Table 2). We used the Seurat 4.3 *R* package [48] to preprocess and normalize the data, including identifying low-quality samples, calculating the ratio of mitochondrial genes and red blood cell genes, filtering cells that do not meet the requirements, removing batch effect, and data normalization. We then used a UMAP algorithm to reduce dimensionality and visualize the data to show their characteristics. We annotated the different cell subgroups of the clusters using the SingleR 2.2 *R* package [49] and selected specific cell subgroups for downstream analysis based on the changes in various cell subgroups between samples and genders.

### 4.2. GO and KEGG Analyses of DEGs in Macrophages Between Male and Female PAH Samples

We screened out DEGs in macrophages between male and female PAH samples using the FindMarkers function and displayed the volcano plot of gene expression using the ggplot2 *R* package. We used the DAVID (https://david.ncifcrf.gov/) [50] tools to analyze the biological process (BP) and signal pathways of the DEGs in macrophages between male and female PAH samples. All BP terms and KEGG pathways with a *p* value < 0.05 were considered statistically significant.

### 4.3. Comparison of Macrophage Subgroups Ratio Between Male and Female PAH Samples

We analyzed the ratio of M1 and M2 macrophages in male and female PAH samples by extracting macrophage datasets from single-cell sequencing data. Seurat and SingleR *R* packages were used for the analysis. The cell markers of M1 macrophages were CD68, CD74, CD86, TSPO, CCL2, PTGS2, CCL3, INHBA, and NOS2, and the cell markers of M2 macrophages were CD68, CD74, CD163, CD16, CCL8, FOLR2, C1QA, TERM2, and MRC1. We used the FindMarkers function to screen out DEGs in M1 macrophages between male and female PAH samples. GO and KEGG analyses were performed on the DEGs as described above.

### 4.4. Isolation and Culture of Rat Lung Macrophages Cells

After anesthesia, the lung tissues were removed from the mice, and the trachea was clamped so that the lungs were suspended. PBS was injected into the trachea to flush the alveoli. After alveolar macrophages were removed, the trachea was cut off, and the lung tissues were placed in a flat dish in the precooled RPMI-1640 culture solution, and the lung tissues were separated on ice until the lung tissues were completely separated from the bronchial tree. The lung tissue was then passed through a wire mesh to separate individual cells. The cells were cultured in 6-well plates with 1–2 mL of complete medium RPMI-1640 for 2 h, and the adherent cells were washed and continued to be cultured with fresh complete medium for subsequent studies.

### 4.5. shRNA Design and Transfection

The shRNA against RPL39 was synthesized by GeneChem (Wuhan, China) and the sense sequences of shRNA were 5′-TTCAAACTGATGTTCTGGTT-3′. The nontargeted normal shRNA sequences were 5′-TTCTCCGAACGTGTCACGT-3′. Macrophages were cultured in a 6 cm cell culture dish at 60%–80% confluence and transfected with shRNA plasmid using a lentivirus vector. We incubated the mixture at 37°C for 48 h and then harvested the cells to experiment as needed.

### 4.6. Western Blot

Proteins extracted from macrophages were separated by SDS–PAGE and transferred onto polyvinylidene fluoride membranes. The membranes were blocked with 5% skim milk at room temperature for 1 h and then incubated with RPL39 (1:1000, ab74740, Abcam) and *β*-actin (1:10000, 66009-1-LG, Proteintech) at 4°C overnight. After three washes with TBST buffer, the membranes were incubated with horseradish peroxidase–conjugated secondary antibodies and enhanced chemiluminescence reagents.

### 4.7. Cell Counting Kit-8 (CCK-8) Determination of Macrophages

CCK-8 was used to detect the effect of shRNA on the macrophages proliferation; the macrophages with 70%–80% confluency were starved in serum-free RPMI-1640 medium for 24 h, then replaced by RPMI-1640 medium containing 10% fetal bovine serum after starvation, and 10 μL of CCK-8 was added to each well for 3 h. The absorbance (a) value at 450 nm was measured to detect cell proliferation.

### 4.8. Animals and Treatments

Ten male and 10 female Sprague Dawley rats (Hunan Silaike Jingda Laboratory Animal Co. LTD) were divided into normal and shRNA groups, which were transfected with vector and shRNA plasmids by airway administration, respectively. After 72 h of dosing, all rats were injected intraperitoneally with monocrotaline (MCT) (Sigma:315-22-0) at a dose of 60 mg/kg. Three weeks later, we successfully established the animal disease model of PAH. All animal experiments were approved by the Ethical Committee of Shihezi University (A2023-006-01).

### 4.9. Hemodynamic Analysis

After the rats were anesthetized, the jugular vein was bluntly separated, a small opening was cut in the jugular vein, and a PE-50 catheter (0.6 mm inside diameter and 0.9 mm outside diameter) was inserted. The PowerLab system was connected to measure the mPAP. The chest was then opened and a Millar catheter was inserted into the right ventricle, and CO, ventricular pressure, EF, and ventricular volume were measured.

### 4.10. Statistical Methods

Data in the box plots are presented as mean ± SD and analyzed by 2-way ANOVA multiple comparisons, and all experiments were successfully repeated five times and yielded consistent experimental results. The *p* value adjusted by Bonferroni was used to determine whether the difference between groups was statistically significant. ⁣^∗^*p* < 0.05, ⁣^∗∗^*p* < 0.01, ⁣^∗∗∗^*p* < 0.001, and ⁣^∗∗∗∗^*p* < 0.0001.

## Figures and Tables

**Table 1:** GO and KEGG results of DEGs in M1 macrophages between male and female PAH samples.

Category	Term	Count	*p* value
KEGG	Phagosome	14	3.90E − 07
KEGG	Chemokine signaling pathway	12	1.40E − 04
KEGG	Cytokine–cytokine receptor interaction	12	4.90E − 03
KEGG	IL-17 signaling pathway	11	1.30E − 06
KEGG	NF-kappa B signaling pathway	11	3.20E − 06
KEGG	Toll-like receptor signaling pathway	11	3.20E − 06
KEGG	Osteoclast differentiation	11	2.10E − 05
KEGG	Lysosome	11	2.70E − 05
KEGG	Ribosome	11	2.00E − 04
BP	Inflammatory response	22	1.30E − 09
BP	Immune response	23	3.50E − 09
BP	Positive regulation of ERK1 and ERK2 cascade	15	4.60E − 08
BP	Neutrophil chemotaxis	10	1.00E − 07
BP	Antigen processing and presentation of exogenous peptide antigen via MHC Class II	7	5.80E − 07
BP	Cellular response to tumor necrosis factor	11	9.50E − 07
BP	Apoptotic process	21	2.60E − 06
BP	Cytoplasmic translation	9	2.70E − 06
BP	Chemokine-mediated signaling pathway	8	5.30E − 06
BP	Positive regulation of nitric oxide biosynthetic process	7	5.30E − 06

**Table 2:** Single-cell sample details in GSE169471 and GSE228644.

Dataset	Sample size	Type	ID	Age	Gender
GSE169471	11	PAH	GSM5206779	21	Male
		PAH	GSM5206780	50	Female
		PAH	GSM5206781	36	Female

GSE228644	13	PAH	GSM6424658	37	Male
		PAH	GSM6424659	52	Female
		PAH	GSM6424660	63	Male

## Data Availability

The data that support the findings of this study are available from the corresponding author upon reasonable request.
